# Examining subjective cognitive decline in a Canadian multi‐ethnic cohort of older adults

**DOI:** 10.1002/alz70857_098783

**Published:** 2025-12-24

**Authors:** Georgia Gopinath, Rachel Yep, Tulip Marawi, Rohina Kumar, Simran Malhotra, Angelina Zhang, Madeline Wood Alexander, Alexander J Nyman, Silina Z Boshmaf, Katie L Vandeloo, Sandra E. Black, Maged Goubran, Jennifer S Rabin

**Affiliations:** ^1^ Hurvitz Brain Sciences Program, Sunnybrook Research Institute, Toronto, ON, Canada; ^2^ Rehabilitation Sciences Institute, University of Toronto, Toronto, ON, Canada; ^3^ University of Toronto, Toronto, ON, Canada; ^4^ Hurvitz Brain Sciences Program, Toronto, ON, Canada; ^5^ Division of Neurology, Department of Medicine, University of Toronto, Toronto, ON, Canada; ^6^ Department of Medical Biophysics, University of Toronto, Toronto, ON, Canada; ^7^ Harquail Centre for Neuromodulation, Sunnybrook Research Institute, Toronto, ON, Canada

## Abstract

**Background:**

Subjective cognitive decline (SCD) may represent one of the earliest symptoms of Alzheimer's disease. However, SCD remains understudied in diverse ethnoracial groups, particularly among individuals of Asian descent. Leveraging data from a cohort study of Canadian South Asian, Chinese, and White older adults, this study investigated ethnic differences in: (1) SCD burden, (2) the association between self‐ and study partner‐reported SCD, and (3) the demographic and neuropsychiatric factors associated with SCD.

**Methods:**

Participants were South Asian, Chinese, and White adults aged 55‐85 enrolled in the observational cohort study, *Ca* nadian *M* ulti‐ *E* thnic *R* esearch on *A* ging (CAMERA). Participants were classified as cognitively unimpaired based on a Clinical Dementia Rating global score of 0. SCD was assessed using the self‐ and study partner‐reported Cognitive Functioning Index (CFI). Depression and anxiety symptoms were measured using the Geriatric Depression Scale and Generalized Anxiety Disorder‐7 Scale, respectively. Linear regression models were used to examine associations of interest, adjusting for age, sex, years of education, as well as depression and anxiety symptoms.

**Results:**

We included 140 participants (mean age=65.8±6.4, 69.3% female) who self‐identified as South Asian (*n* = 38), Chinese (*n* = 52) or White (*n* = 50). Self‐reported CFI scores were significantly higher in South Asian (β=0.9, *p* = 0.02) and Chinese participants (β=1.4, *p* < 0.001) compared to White participants, and did not significantly differ between South Asian and Chinese participants (β=0.5, *p* = 0.2). Self‐ and study partner‐reported CFI scores were significantly associated across the whole sample (β=0.3, *p* = 0.01), with no moderation by ethnicity (*p*>0.05). However, the associations between symptoms of depression and anxiety with self‐reported CFI scores were stronger in South Asian and Chinese participants compared to White participants (*p* < 0.05). The associations between depression and anxiety with CFI did not significantly differ between South Asian and Chinese participants (*p*>0.05).

**Conclusions:**

In a sample of cognitively unimpaired older adults, self‐ and study partner‐reported SCD were associated, and the strength of the association did not differ across ethnic groups. However, among South Asian and Chinese participants, SCD burden was greater and more strongly associated with depression and anxiety symptoms. These findings underscore the importance of considering ethnoracial differences and neuropsychiatric symptoms when assessing SCD in diverse populations.

